# Amino acids as nutritional factors and (p)ppGpp as an alarmone of the stringent response regulate natural transformation in *Micrococcus luteus*

**DOI:** 10.1038/s41598-019-47423-x

**Published:** 2019-07-30

**Authors:** Antoni Lichev, Angel Angelov, Inigo Cucurull, Wolfgang Liebl

**Affiliations:** Chair of Microbiology, Technical University of Munich, Freising-Weihenstephan, Germany

**Keywords:** Bacterial development, Bacterial genetics, Microbiology, Gene regulation

## Abstract

Natural competence for genetic transformation refers to the natural ability of various bacteria to take up exogenous DNA from their surroundings and to incorporate internalized genetic information into their genomes. By promoting bacterial diversification and adaptability, this process represents a major driving force in bacterial evolution. *Micrococcus luteus* was one of the first organisms used to study natural transformation in bacteria. Since then, however, only very little information about this phenomenon has been reported in *M*. *luteus* or in any member of the *Actinobacteria* phylum (low-GC Gram-positive bacteria). Previous work in our group indicated major differences between the transformation apparatus of *M*. *luteus* and the transformation machinery described for various Gram-negative and Gram-positive model bacteria belonging to the phyla *Proteobacteria* and *Firmicutes* (high-GC Gram-positive bacteria). This prompted us to initiate a study concerning the regulation mechanism of competence development in *M*. *luteus*. In this report, we identify amino acids as a nutritional factor that influences competence in a concentration-dependent manner. By using a transcriptional reporter strain for one of the late competence genes, we demonstrate how increasing concentrations of both amino acids mixtures and single amino acids supplemented to the growth medium affect transformability on transcriptional and post-transcriptional level. Furthermore, we revisit previously generated auxotrophic mutants to show that the transformation machinery is turned down during a state of extreme hunger for amino acids presumably as a part of a general response to auxotrophy. Finally, by generating and analysing knockout mutants for two predicted stringent response enzymes, we provide evidence for the involvement of the alarmone (p)ppGpp as a putative mediator of the effects on transformation development caused by amino acids. As a member of the *Actinobacteria* phylum, *M*. *luteus* could serve as a model for other representatives of the phylum, including a number of important human pathogens.

## Introduction

Natural competence for genetic transformation is widely spread throughout the main phylogenetic taxa and is not a rare phenomenon^1^. It denotes the ability of bacteria to uptake and internalize exogenous, “naked” DNA from their surroundings and to incorporate it into their chromosomes *via* homologous recombination. Together with transduction and conjugation, transformation represents one of the main routes for lateral gene transfer and as such is considered to be a key driver of rapid bacterial evolution^2^.

By promoting genome plasticity and adaptability to inhospitable environments, natural transformation has contributed to bacterial diversification and has shaped large portions of bacterial genomes by allowing microorganisms to transfer genetic material between major bacterial lineages^2^. In recent years, due to the increasing evidence for the involvement of this process in the dissemination of antibiotic resistances, virulence traits^2^, and vaccine evasion mechanisms^3^, researchers have found a new incentive to study the molecular mechanics driving competence development. For this reason, we initiated an investigation into the regulation of natural transformation in *M*. *luteus* - a member of the *Actinobacteria* phylum, which also contains a number of important human pathogens, and for which detailed knowledge about the factors affecting competence development is missing.

Historically, *M*. *luteus* was one of the first organisms used to study natural transformation in bacteria^4–6^. However, in the past decades mainly the high-GC Gram-positive bacteria (phylum *Firmicutes*) *Bacillus subtilis* and *Streptococcus pneumoniae*, and various Gram-negative bacteria (*Neisseria gonorrhoeae*, *Haemophilus influenzae*, *Vibrio cholerae*, *Helicobacter pylori*, *Thermus thermophilus*) served as the main model organisms to study natural transformation. Even though depending on their cell wall bacteria need to overcome different obstacles during DNA uptake and transport across the cellular envelope, nearly all transformable species seem to have evolved common structures and mechanisms to facilitate genetic competence^1^. Briefly, all characterized systems require the presence of a putative transformation (pseudo-)pilus, a DNA receptor (in *B*. *subtilis*, encoded by the gene *comEA*), a transmembrane pore (in *B*. *subtilis*, encoded by the gene *comEC*), as well as proteins involved in the homologous recombination of the DNA^1^. For more detailed information on the topic, we refer to the reviews by Dubnau^7^, Johnston *et al*.^1^, and Chen and Dubnau^8,9^. In terms of regulatory pathways and induction of competence, however, bacteria seem to have evolved divergent regulatory systems^1,10–12^. Since the state of competence is a complex, multifactorial trait, many different levels of regulation have emerged. As an example, the control on transcriptional level involves alternative sigma factors (e.g. σ^X^ in *S*. *pneumoniae*), transcription activators (e.g. ComK in *B*. *subtilis*), transcription co-regulators (e.g. TfoX in *V*. *cholera*), and, additionally, proteins regulating the activity of these regulators. Translational and post-translational control via non-coding small RNAs^13^ or Clp proteases^14,15^ have also been documented in different bacteria. Importantly, most regulatory pathways vary significantly among transformable species, so no general rule that governs competence development exists. Notable dissimilarities in the growth phase dependencies, the inducing environmental cues, and the cellular triggers of natural transformation also contribute to the complexity of the topic^1^. While some organisms reach the peak of transformability in the stationary growth phase, others develop competence during the early exponential phase. Some bacteria such as *B*. *subtilis* and *H*. *influenzae* respond to nutritional signals^11,16^, others, like *S*. *pneumoniae* and *Legionella pneumophila* sense varying stress conditions to trigger entry in the transformable state^17,18^. Intriguingly, up to date we are familiar with only one study (conducted in 1968 on *B*. *subtilis*) that provides a hint about the regulatory function of amino acids in the context of natural transformation^19^.

The stringent response refers to a very widely distributed physiological response of bacteria to nutrient deprivation, heat shock or other stress factors^20^. Usually, it is accompanied by a massive switch in the transcription profile aimed to steer the cellular metabolism away from ribosome synthesis, DNA replication and production of membrane components and towards the upkeep of general metabolism, glycolysis, amino acid synthesis^21^, and other basic survival functions. The driving force of the stringent response is the alarmone (p)ppGpp that can exert its effects not only on transcriptional level by regulating the expression of many different genes, but also by direct binding to certain enzymes or by indirect modulation of intracellular GTP levels^20^. Since the action of the alarmone is concentration-dependent, several enzyme classes are involved in the fine-tuning of its intracellular pool^20^, including the RelA-SpoT homologue (RSH) family of bifunctional proteins that can both synthesize and degrade (p)ppGpp^22,23^, Nudix hydrolases^24,25^, certain GTPases^26^ and the only recently discovered small alarmone synthetases (SASs) and hydrolases (SAHs)^27–29^.

Up to date, the stringent response has mainly been implicated in bacterial growth^20^, virulence^30^, survival during host invasion^31^, antibiotic resistance^32^ and persistence^33^. To the best of our knowledge, only a few studies have reported a connection between the stringent response and natural transformation. The information about such a link comes primarily from studies conducted on the oral pathogen *Streptococcus mutans*^34–36^ as well as on the model organism *Bacillus subtilis* where a (p)ppGpp dependant change in the intracellular GTP concentration modulates the activity of the master regulator CodY^37^. Among others, CodY negatively regulates ComK known as the key factor for competence development in *B*. *subtilis*^37^. Importantly, so far our group has not been able to identify any homologs to CodY or ComK in *M*. *luteus*.

Since *Actinobacteria* are very poorly represented in comprehensive reviews covering this topic, we decided to investigate the conditions under which *M*. *luteus* develops competence. In a previous study, our group has demonstrated that natural transformation in *M*. *luteus* apparently utilizes a different (pseudo)pilus structure than the usually found type IV (pseudo)pili and that competence is a transient regulated trait that is affected by the growth phase and the nutritional state of the bacteria^38^. In this study, we pinpoint amino acids as modulators of competence development in this organism and we identify them as the nutritional signal that serves as a trigger of competence. We provide evidence that under auxotrophic conditions cells “turn off” the transformation apparatus. Finally, we were curious to understand the mechanism of how *M*. *luteus* senses amino acids and responds to different amino acid concentrations by modulating its transformability. Here, we come to the conclusion that presumably the stringent response plays an important role in regulating competence since no homologs of the typical master regulators of other competent bacteria have been identified so far.

## Results

### Amino acids in the growth medium serve as a nutritional signal regulating competence development in a concentration-dependent manner

In a previous study, we have demonstrated that competence development in *Micrococcus luteus* trpE16 is dependent on the nutritional state of the bacteria^38^. Cells grown in complex media such as LB display approximately 1000-fold lower transformation frequencies than those grown in defined minimal medium (MM). Diluting complex media also positively affects transformability so that cells grown in diluted LB can reach transformation frequencies comparable to those of cells grown in MM^38^. This data strongly indicates that nutrient limitation induces competence development. However, since no information about the specific trigger of competence in MM is available, we initiated a study to try and pinpoint the nutritional factor that modulates natural transformation in *M*. *luteus*.

Sezonov *et al*. have demonstrated that the preferred carbon sources for *E*. *coli* in LB medium are catabolizable amino acids, even in the presence of sugars^39^. Since the defined minimal medium used in our study does not contain any amino acids (other than glutamate and tryptophan)^40^, we assumed that amino acid starvation in MM or in diluted LB could be the signal triggering competence development in *M*. *luteus*. To investigate this, we performed a transformation frequency assay with cells grown in LB, MM, or MM to which a mixture of 15 amino acids at the concentrations listed in Table 1 was added. In addition, we inoculated cells in MM supplemented with increasing concentrations of peptone and performed again transformation frequency assays at different time points. Peptone supplementation varied between 0.1 g/l and 10 g/l with the highest concentration being equal to that of LB medium.

**Table 1:** Composition of the amino acids mixture used in this study according to Wolin and Naylor^40^.

Amino acid	Manufacturer	Final concentration
L-alanine	Formedium	0.65 mg/ml
L-arginine	Formedium	0.65 mg/ml
L-cysteine	Formedium	0.2 mg/ml
L-glutamic acid	Formedium	0.65 mg/ml
Glycine	Formedium	0.35 mg/ml
L-histidine	Formedium	0.35 mg/ml
L-lysine	Formedium	0.35 mg/ml
L-methionine	Formedium	0.35 mg/ml
L-phenylalanine	Formedium	0.35 mg/ml
L-proline	Formedium	0.35 mg/ml
L-serine	Formedium	0.2 mg/ml
L-tryptophan	Formedium	0.2 mg/ml
L-valine	Formedium	0.65 mg/ml
L-isoleucine	Formedium	0.65 mg/ml
L-leucine	Formedium	0.65 mg/ml
Total:		**6**.**6 mg/ml**

**Figure 1:**
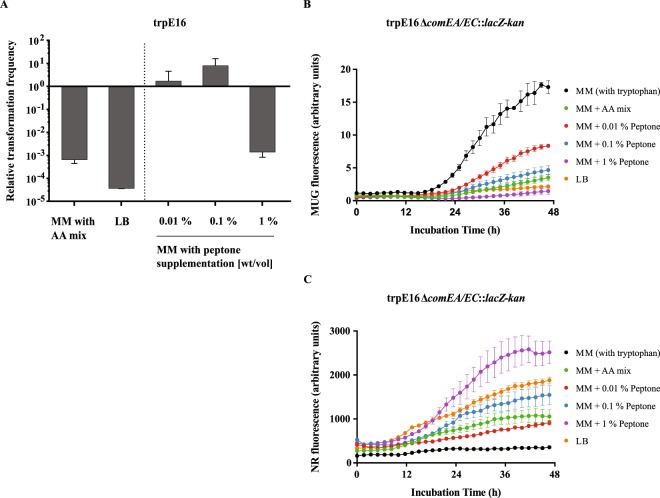
Regulation of transformability by supplementation with amino acids mixtures. (**A**) A mixture of 15 amino acids at a total concentration of 6.6 mg/ml (Table 1) added to the MM suppressed transformability of *M*. *luteus* almost to the levels measured in complete medium (LB). The effect of peptone supplementation on the transformation frequency was concentration-dependent and bidirectional. A concentration comparable to the peptone concentration of LB medium inhibited transformability, whereas lower concentrations lead to a slight increase. Bars represent the mean transformation frequencies (n = 4) relative to the mean transformation frequency of the reference strain *M*. *luteus* trpE16 grown in MM for 20 hours, which was set to 1. The error bars indicate the SD of the biological replicates. (**B**,**C**) Transcriptional reporter assay for the activity of the promoter of *comEA/EC* under different conditions. A transcriptional reporter strain (Δ*comEA*/*EC*:*lacZ*) expressing *lacZ* from the native *comEA/EC* promoter was incubated in media containing different amino acids mixtures and a fluorogenic LacZ substrate (MUG). Fluorescence was measured every 10 minutes for 96 hours. Due to substrate depletion, only the first 48 hours are presented. Nile red was utilized to monitor bacterial growth (**C**) and was used to normalize the MUG signal (**B**). Each data point represents the mean of three separate measurements. The error bars represent the standard error of the mean (SEM). Method validation is described in Supplementary Information.

Expectedly, after 20 hours of incubation, the transformation frequency of the LB culture was approximately 4 log10 scales lower than that of the MM culture (Fig. 1A). An almost just as strong decrease of transformability was also measured in case of the cells grown in MM supplemented with amino acids. The absolute transformation frequency of those cells reached 1 × 10^−6^. The 10-fold difference relative to the cells grown in LB could possibly be attributed to the absence of the amino acids L-asparagine, L-aspartic acid, L-glutamine, L-threonine and L-tyrosine from the used amino acids mixture. In the case of the cells grown in peptone-supplemented MM, low concentrations of peptone (0.01% − 0.1%) caused a slight increase in transformability relative to the non-supplemented medium (Fig. 1A). However, the observed change was very small and could also fall within the standard error of the assay. In contrast, the supplementation with 10 g/l peptone led to a 1000-fold decrease in the transformability of *M*. *luteus*, similar to the effect of full medium or of supplementation with a mixture of amino acids. Surprisingly, a stepwise increase in the peptone concentration did not result in a corresponding stepwise change in transformability. This data suggests that only when a certain threshold concentration of amino acids or metabolizable peptides in the growth medium is reached does the transformation frequency decrease in an abrupt manner. Furthermore, the result of our experiment supports the hypothesis that lack of amino acids is the nutritional signal that triggers competence development in MM and that reciprocally, their abundance in LB exerts a suppressing effect.

To get a better understanding of the transcriptional response of the cells under different supplementations, we used the *M*. *luteus* transcriptional reporter strain Δ*comEA*/*EC*:*lacZ* that expresses the full-length *E*.*coli lacZ* gene from the native *M*. *luteus comEA/EC* promoter. Incubation of this mutant strain in differently supplemented growth media together with 100 µg/ml 4-methylumbelliferyl-β-D-galactopyranoside (MUG) as a fluorogenic LacZ substrate allowed for a kinetic study of the activity of the promoter. Nile red at a concentration of 20 µg/ml was used to monitor growth, to normalize the signal and to account for variations in the growth rate (Fig. 1C). The results depicted in Fig. 1B revealed a clear connection between the activity of the promoter and the concentration of amino acids in the growth medium. Cells incubated in pure MM produced the strongest signal, whereas bacteria grown in LB or in MM supplemented with 1% peptone (wt/vol) demonstrated a relatively strong repression of the promoter. The growth experiments with less added peptone (1 g/l or 0.1 g/l) or with an added amino acids mixture (6.6 g/l) also confirmed the observation that higher amounts of amino acids correlate with a decreased promoter activity. However, the transcription marker expression data differed from the results of the transformation frequency assays (see above) by revealing a stepwise decrease in reporter activity with increasing amino acid concentrations. This, along with the fact that low peptone concentrations did not reduce the transformability of the cells (Fig. 1A) in spite of the repressed *comEA/EC* promoter under these conditions (Fig. 1B), indicated that competence development is a complex multifactorial trait regulated at several different levels.

### Auxotrophy inhibits competence development on the transcriptional level

**Figure 2:**
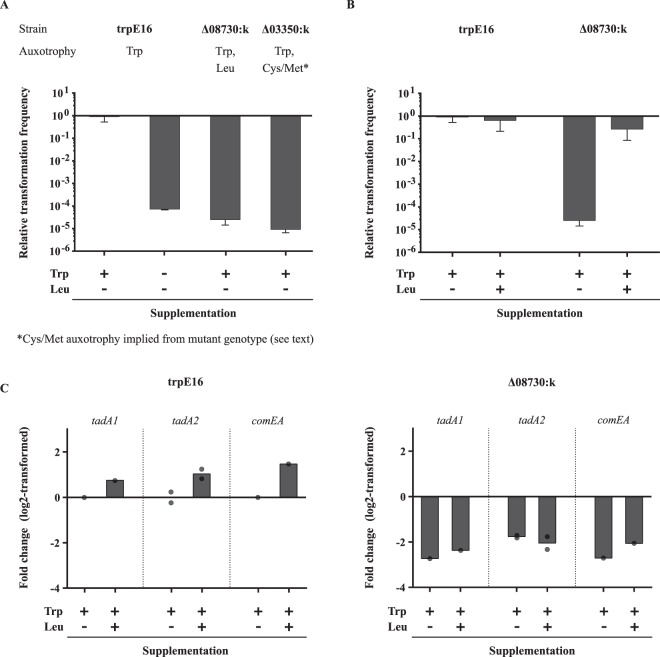
Transformation frequency and expression of competence-related genes under auxotrophic conditions. (**A**) Transformation frequency of different auxotrophic strains strongly decreased after 20 hours of growth in MM. (**B**) Supplementation of MM with 5 mM leucine restored transformability in the leucine auxotrophic strain ∆08730:k. (A and B) Bars represent the mean transformation frequencies [n = 2 for (A), n = 6 for (**B**)] relative to the mean transformation frequency of the *M*. *luteus* trpE16 reference strain grown in MM for 20 hours which was set to 1. The error bars indicate the SD of the biological replicates. (**C**) Relative gene expression levels (qRT-PCR) of the competence-related genes *tadA1* (Mlut_07500), *tadA2* (Mlut_01819) and *comEA* (Mlut_12450) in the reference strain trpE16 (left) and the leucine auxotrophic mutant strain ∆08730:k (right) of *M*. *luteus*. RNA was isolated from the same cultures and at the same time points for which transformability was determined. The bars represent the log2-transformed mean fold change of the biological replicates relative to the wild-type strain grown in MM. The dots represent the measured values of each biological replicate. The _∆∆_C_t_ method was used for data evaluation^65^.

**Figure 3:**
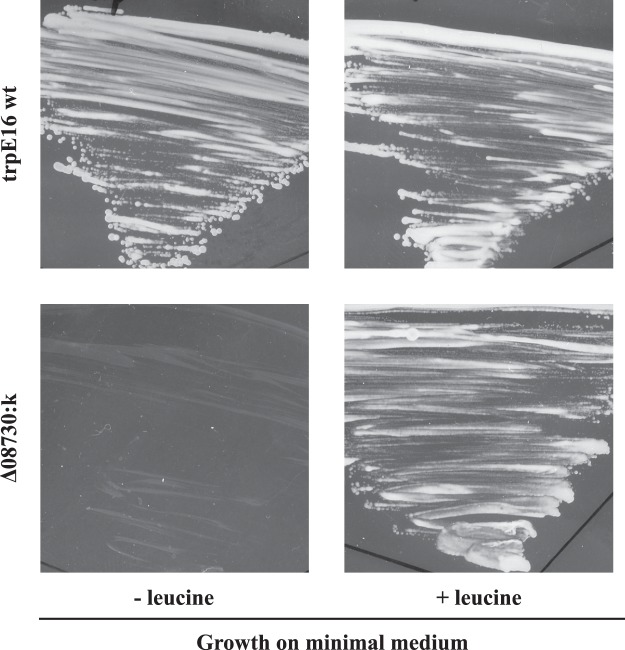
Growth of trpE16 and ∆08730:k on MM plates supplemented with (+) or without (−) 5 mM leucine. The reference strain of *M*. *luteus* (trpE16) and the mutant strain ∆08730:k were streaked on tryptophan-containing MM plates with or without leucine. ∆08730:k was unable to grow without leucine supplementation indicating that the deletion of Mlut_08730 caused an auxotrophy for this amino acid.

In recent work, we systematically deleted genes for predicted DNA binding proteins in *M*. *luteus*^41^. In this gene deletion library, we identified two mutants (∆08730:k and ∆03350:k) with dramatically decreased transformability (Fig. 2A). Mlut_08730 is a gene encoding a transcriptional regulator of the IclR family, which is known to regulate the expression of antibiotic resistance and amino acid biosynthesis genes in different organisms^42,43^. RNA-Seq experiments with the ∆08730:k mutant grown in MM revealed that in this strain there is a strong downregulation of the genes encoding the large and small subunits of the 3-isopropylmalate dehydratase. 3-isopropylmalate dehydratase is the enzyme responsible for the conversion of 2-isopropylmalate into 3-isopropylmalate and is therefore crucial for leucine production. Growth experiments with leucine supplementation showed that the ∆08730:k mutant indeed is auxotrophic for leucine (Fig. 3). Analogously, we constructed the deletion strain ∆03350:k that exhibits an impaired cysteine and/or methionine metabolism. Mlut_03350 is a response regulator with a CheY-like receiver domain and a DNA binding domain and is possibly involved in the regulation of the genes responsible for the biosynthesis of the two amino acids. RNA sequencing revealed a strong downregulation of several gene clusters implicated in cysteine and methionine production^44^ and high genome-wide similarity in the transcription profile of this strain in comparison to the leucine auxotroph. Growth experiments on MM plates to confirm the predicted Met/Cys auxotrophy were not conclusive due to an inhibiting effect of supplemented cysteine on the growth of the parental strain that served as a reference.

Transformation frequency assays were carried out with the parental strain (strain trpE16), which carries a known point mutation in the *trpE* gene and therefore is tryptophan auxotrophic, and with the deletion mutants trpE16∆08730:k and trpE16∆03350:k grown in MM for 20 hours under auxotrophic conditions (i.e. without tryptophan supplementation for the strain trpE16, with tryptophan supplementation for strains trpE16∆08730:k and trpE16∆03350:k). Importantly, even though the bacteria are not able to grow unlimitedly under auxotrophic conditions and prolonged cultivation eventually lead to growth arrest, based on the increase in the optical density of the bacterial cultures, they still underwent 1 to 2 cell divisions within the first 20 hours of incubation. This was comparable to the growth of the reference strain grown under prototrophic conditions (strain trpE16 in MM with tryptophan supplementation). Regardless of the auxotrophy, during this time all tested strains reached an OD_600_ of around 0.8 after having been inoculated at an OD_600_ of 0.2. However, such growth of the three strains under auxotrophic conditions inhibited competence development to a great extent (Fig. 2A). In all three cases, the measured transformation frequency was at least four log10 scales lower than that of the trpE16 reference strain grown in MM with tryptophan supplementation. Independent from the particular amino acid auxotrophy, the absolute transformation frequencies of these strains varied between 1 × 10^−7^ and 1 × 10^−9^ and in some instances even were below the detection limit of the assay (1 × 10^−9^).

Next, we performed the transformation frequency assay with the ∆08730:k strain but this time the MM (containing tryptophan due to the tryptophan auxotrophy of the parental reference strain trpE16) was supplemented with 5 mM leucine to test whether or not complementation of the mutant’s auxotrophy would restore transformability. Indeed, our observations showed an increase in the transformation frequency of the leucine auxotroph back to wild-type level (Fig. 2B). Thus, judging from the results obtained with the tryptophan-auxotrophic strain trpE16 in the absence of tryptophan or with the leucine-auxotrophic strain ∆08730:k in the absence of leucine, the inability to synthesize one amino acid apparently is sufficient to cause a strong repression of competence development in *M*. *luteus* unless the respective amino acid is supplied externally.

A rational explanation of the observed phenotype could be the inefficient translation of the transcripts encoding the proteins necessary for the assembly of the transformation machinery. To address the issue if the auxotrophic conditions also had an effect on the transcription of the competence-related genes, we performed qRT-PCR on *tadA1* (Mlut_07500), *tadA2* (Mlut_01819), and *comEA* (Mlut_12450), which encode two type IV pilus assembly ATPases TadA1 and TadA2, as well as the membrane-bound DNA receptor ComEA, required for binding and internalization of the transforming DNA during transformation^38^. Here, mRNA was isolated from the same cells and at the same time points for which transformability was determined. A 4-fold decrease in expression of all three investigated genes was observed in the leucine auxotrophic ∆08730:k strain (Fig. 2C), indicating that during amino acid starvation *M*. *luteus* turns down the expression of the transformation apparatus and possibly redirects its resources towards stress survival.

Remarkably, complementation of the leucine auxotrophic ∆08730:k mutant by supplementing the growth medium with 5 mM leucine did not affect the transcription of the competence-related genes. In the trpE16 genetic background, external leucine even exerted a modest positive effect on transcription of these genes (Fig. 2C) but in the ∆08730:k strain, the genes were downregulated and remained downregulated upon leucine supplementation (Fig. 2C) even though the transformability was reverted back to wild-type level (Fig. 2B). This strong discrepancy between the two phenotypes, i.e., transcriptional regulation of the competence-related genes on one hand and transformation frequency under leucine deficiency *vs* leucine supplementation conditions on the other hand, supports the hypothesis that cells either discriminate between externally supplemented and self-produced amino acids in their metabolic response, or the deleted transcription factor IclR is directly involved in the regulation of the expression of the competence-related genes. To this end, further investigation of the details of this regulation is still necessary.

### Modulatory function of amino acids in the transcriptional control of competence development

**Figure 4:**
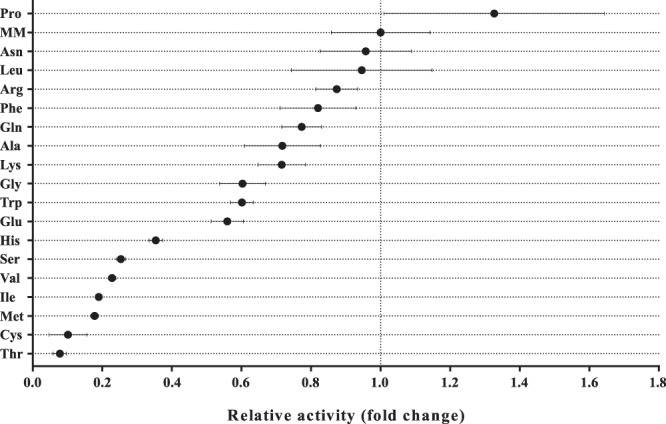
Transcriptional effect of different amino acids on the expression at the promoter of *comEA/EC*. The transcriptional reporter strain Δ*comEA*/*EC*:*lacZ* was incubated in MM supplemented with 5 mM of each of the designated amino acids. Kinetic data for the promoter activity was recorded over 96 hours by measuring the fluorescence generated from the cleavage of the LacZ substrate MUG. The fluorescence signals of the MUG assays were normalized by the fluorescence signal of Nile red used as a proxy for growth. A four parameter logistic model was fitted to the kinetic curves (n = 3). The slope of the logistic curve served as a measure for the relative promoter activity (logistic model equation and additional data are present in Supplementary Information). The fold-change in relative promoter activity caused by each amino acid in comparison to the non-supplemented MM is shown. The bars represent the standard error of the fitted curves.

After being able to identify amino acids as modulators of competence in *M*. *luteus*, we addressed the issue of whether or not the bacterial cells can discriminate between them. It remained unclear from our initial experiments if the cellular response towards amino acids in the growth medium was specific or rather general and purely concentration-dependent. To shed light on this question, we again used the transcriptional reporter strain trpE16 Δ*comEA*/*EC*:*lacZ* to investigate the role of each individual amino acid listed in Table 1 in competence development. Intriguingly, with each supplementation, the bacteria behaved differently in terms of expression at the *comEA/EC* promoter (Fig. 4). In comparison to the non-supplemented MM, almost all amino acids resulted in a shift in the relative promoter activity which was determined as the slope of a logistic model fitted to the measured kinetic curves. The relative promoter activities spanned an about 17-fold range between the lowest (with Thr) and highest activity (with Pro). Even though the induced change was very small for most of the amino acids (fold change of +/− 0.5), histidine, serine, valine, isoleucine, methionine, cysteine, and threonine caused strong repression of the promoter. It is important to mention that some of these repressing amino acids interfered with the growth rate of *M*. *luteus* in MM, possibly due to negative feedback loops in the biosynthesis of the amino acids^45^ and/or to impaired catabolic pathways. However, since we used Nile red staining to normalize the fluorescence signal and to account for fluctuations in the growth rate, we consider the values depicted in Fig. 4 to be actual repression. Unexpectedly, proline demonstrated a slight inducing effect on the promoter of *comEA/EC*. Still, it is very unlikely that such a relatively small shift measured on the transcriptional level would have a great impact on the overall transformability of the cells. More importantly, though, the results from this experiment indicate that the bacteria do not respond equally to each amino acid in terms of competence development, as would be expected if they serve solely as a nutrition source.

After observing the transcriptional effect of some amino acids on the activity of the competence-related *comEA/EC* promoter, we decided to test if they could also modulate the overall transformability of *M*. *luteus*. Since branched-chain amino acids (BCAAs) have been implicated in the control of many physiological processes in other bacteria, including competence^46–48^, and since isoleucine and valine proved to be among the strongest transcriptional modifiers in our initial experiment, we decided to focus on the specific role of branched-chain amino acids.

**Figure 5:**
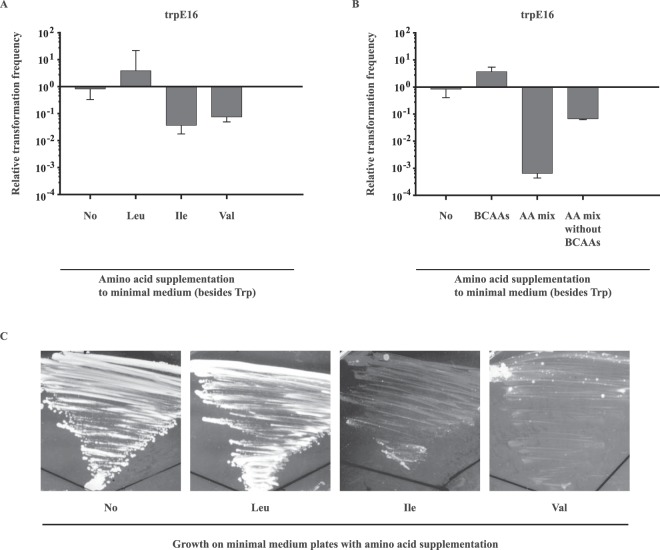
Effect of branched-chain amino acids (BCAAs) on transformability and growth of *M*. *luteus*. (**A**) Modulatory effect of each of the BCAAs on transformability. Each amino acid was supplemented at 0.65 mg/ml. (**B**) Bidirectional effect of a BCAA mixture on transformability. BCAAs added to MM increased the transformation frequency, whereas BCAAs added to MM supplemented with a mixture of other amino acids had an inhibitory effect. (**A**,**B**) Bars represent the mean transformation frequencies (n = 2) relative to the mean transformation frequency of the wild-type strain grown in MM for 20 hours, which was set to 1. The error bars indicate the SD of the biological replicates. (**C**) Growth of *M*. *luteus* on MM plates supplemented with different BCAAs. *M*. *luteus* trpE16 was streaked on MM agar plates with or without supplementation with 5 mM leucine, isoleucine or valine. Plates were grown for seven days at 30 °C.

Initially, transformation frequency assays with cells grown in MM supplemented with leucine, valine or isoleucine at 5 mM each were performed to test if any of the BCAAs would influence transformability. Interestingly, whereas isoleucine and valine caused a modest decrease in transformation frequencies, leucine showed a slightly inducing effect (Fig. 5A). It is important to note once more that both valine and isoleucine inhibited growth in MM (Fig. 5C), and leucine increased the growth rate in MM (Supplementary Fig. S2). However, after 20 hours of incubation, the optical densities of the differently supplemented cultures were comparable and indicated 1 to 2 cell divisions for all cultures before the assay was performed. Still, in spite of the modest extent of the generated effect, this data was in agreement with the transcription data (Fig. 4) and provided a hint that the ability for transformation in *M*. *luteus* is specifically influenced by different BCAAs.

We next performed a transformation frequency assay with cells grown in MM supplemented with either all three BCAAs (at a total concentration of 1.95 mg/ml) or with a mixture of amino acids that lacked BCAAs (4.65 mg/ml). As can be seen from Fig. 5B, the addition of the three branched-chain amino acids to the growth medium caused a slight increase in transformability similar to the addition of leucine alone (Fig. 5A), even though cells grown in MM with isoleucine or valine alone showed a reduction in transformation frequency (Fig. 5A). Control experiments with other combinations of amino acids supplemented at the same concentration as the BCAAs verified the specificity of the response (Supplementary Fig. S3). The transformability of the cells grown in MM supplemented with an amino acids mixture lacking BCAAs indicated that not all amino acids contribute equally to the repressing properties of the amino acids mixture. When the branched-chain amino acids are omitted from the mixture, the transformability was approximately 100-fold less repressed than with the complete mixture. Paradoxically, this data suggests that BCAAs drive the transformation frequency in two opposing directions and can both slightly induce (Fig. 5B, ‘BCAAs’ bar) or significantly contribute to the inhibition of competence (Fig. 5B, ‘AA mix’ bar relative to ‘AA mix without BCAAs’). However, one should take into consideration that in the first case cells grew under starvation conditions due to the lack of amino acids other than the BCAAs, whereas in the second case they were supplemented with a mixture of amino acids.

### Role of intracellular (p)ppGpp production and the stringent response in the regulation of natural transformation in *M*. *luteus*

Our data demonstrated that amino acid limitation can influence competence development in *M*. *luteus*. However, the molecular mechanism behind this regulation remained unclear. Since no homologs of the *B*. *subtilis* pleiotropic repressor CodY have been identified in any *Actinobacteria* member and since the stringent response is one of the alternative cellular responses to amino acid starvation, we decided to examine the possible connection between the two physiological processes and to address the question of how a state of nutrient deprivation translates into a change in transformability.

For this purpose, we first searched the predicted proteome of *M*. *luteus* for proteins with domains similar to those of known RelA-SpoT (RSH) or small alarmone synthetase (SAS) proteins. We identified two distinct loci which code for proteins with the characteristic structure of stringent response enzymes. Mlut_12840 encodes a putative RelA-type protein with both a hydrolase and a synthetase function, as is typical for the members of the *Actinobacteria* phylum^20^. Mlut_12840 contains an HD_4 domain putatively responsible for the phosphohydrolase activity of the enzyme, a RelA_SpoT synthesis domain, and the typical TGS and ACT_4 domains that build the C-terminus of Rel proteins^20^. Mlut_22200 is annotated as a GTP pyrophosphokinase in the BioCyc database and has a single RelA_SpoT synthesis domain. This structure is characteristic for the small alarmone synthetases whose function and underlying mechanism are still not very well studied. We speculated that the gene product of this ORF can, therefore, contribute to the intracellular (p)ppGpp production. Furthermore, we were able to identify 11 proteins containing a NUDIX hydrolase domain that could be involved in the degradation of the alarmone of the stringent response^24,25^.

**Figure 6:**
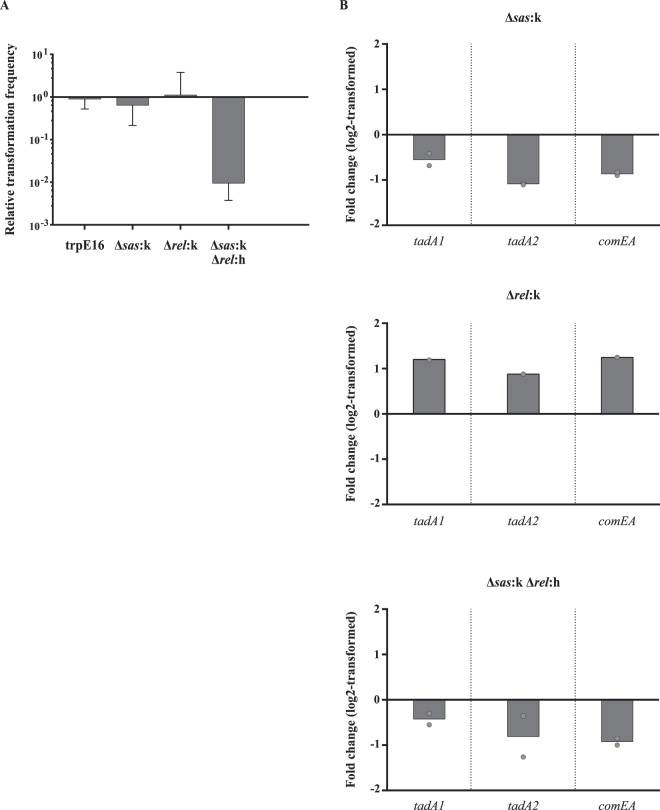
Involvement of the stringent response in natural transformation of *M*. *luteus*. (**A**) Transformation frequency assays of the single and double knockout strains of the genes predicted to encode the small alarmone synthetase as well as the bifunctional Rel protein. An unpaired, two-tailed Student’s t-test showed a difference between the means of the two groups of 0.9883 ± 0.1906 log10 scales (p < 0.0005, n = 6). Bars represent the mean transformation frequencies relative to the mean transformation frequency of the *M*. *luteus* trpE16 reference strain grown in MM for 20 hours which was set to 1. The error bars indicate the SD of the biological replicates. (**B**) qRT-PCR. Relative gene expression levels of the competence-related genes *tadA1* (Mlut_07500), *tadA2* (Mlut_01819) and *comEA* (Mlut_12450) in the stringent response mutant strains ∆*sas*:k (∆*sas::kan*), ∆*rel*:k (∆*rel::kan*) and ∆*sas*:k ∆*rel*:h (∆*sas::kan ∆rel::hyg*) of *M*. *luteus*. RNA was isolated from the same cultures and at the same time points for which transformability was determined. The bars represent the log2-transformed mean fold change of the biological replicates relative to the wild-type strain grown in MM. The dots designate the measured values of each biological replicate. The _∆∆_C_t_ method was used for evaluation of the data^65^.

Deletion mutants for the Mlut_12840 and the Mlut_22200 genetic regions were generated by replacing the ORFs of the genes by a kanamycin resistance cassette, yielding strains designated as ∆*rel*:k and ∆*sas*:k, respectively. Also, a double mutant strain was constructed in which both genes were deleted (∆*sas*:k ∆*rel*:h). Transformation frequency assays were performed with these strains by using cells grown in MM for 20 hours. None of the single mutants showed a significant difference in transformability in comparison to the reference strain trpE16. However, when both genes whose products have a potential (p)ppGpp synthetase function were deleted, a nearly 100-fold drop in the transformation frequency was measured (Fig. 6A). This indicates that the stringent response might be required for the induction of competence in *M*. *luteus* during growth under nutrient limitation such as in a minimal medium. Since transformability was not significantly affected if only one of the enzymes of alarmone metabolism as in the single mutants was missing, apparently such a mutation can be compensated by an alternative enzyme that can complement both a missing synthetase as well as hydrolase function. The severe reduction in transformability in the double mutant where the genes for both RelA_SpoT domain carrying proteins were deleted from the genome of *M*. *luteus* is presumably linked with the strain’s inability to synthesize (p)ppGpp. However, further investigation of the intracellular levels of the alarmone in the different strains and under different conditions is required to study this in more detail.

Additionally, qRT-PCR assays targeting the competence-related genes *tadA1* (Mlut_07500), *tadA2* (Mlut_01819), and *comEA* (Mlut_12450) were once again performed to address the question of how the stringent response affects transformation in *M*. *luteus*. For this, mRNA was isolated from the same cultures and at the same time points for which transformability was determined. Noticeably, none of the mutant strains demonstrated a more than 2-fold change in the relative expression of the investigated genes when compared to the reference strain (Fig. 6B). Even though a 50% repression of gene transcription may be enough to elicit a drop in transformability, the qRT-PCR data from the ∆08730:k strain grown in MM supplemented with leucine suggests that cells can reach wild-type level transformation frequencies even when they exhibit a much stronger repression of the competence-related genes (Fig. 2). Remarkably, the relative expression profile of the three genes we measured does not differ much in the double mutant and in the ∆*sas*:k strain, despite the large differences in the transformability of these strains. All three tested genes were downregulated in both strains. This apparent contradiction between the observed gene expression profiles and the transformability of the mutants may indicate that the stringent response and natural transformation are linked not only on a transcriptional but also on a post-transcriptional level.

**Figure 7:**
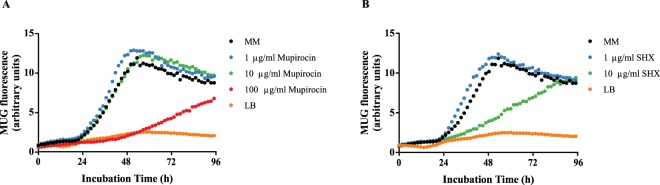
Repression of the competence-related gene *comEA/EC* by the stringent response inducers mupirocin and serine hydroxamate (SHX). The transcriptional reporter strain Δ*comEA*/*EC*:*lacZ* was incubated in LB, MM or MM supplemented with increasing concentrations of either mupirocin or SHX. Kinetic data for the promoter activity was recorded over 96 hours by measuring the fluorescence of the LacZ substrate MUG. The fluorescence signal of MUG was normalized by the fluorescence signal of Nile red used as a proxy for growth. The decrease in the normalized fluorescence signal after 48 hours is the result of substrate depletion. Each data point represents the median of four separate measurements.

To provide additional evidence for the involvement of the stringent response in natural transformation of *M*. *luteus*, we once again used the transcriptional reporter strain Δ*comEA*/*EC*:*lacZ* and kinetically measured the response of the promoter of *comEA/EC* upon incubation of the bacterial cells in a medium containing increasing concentrations of either mupirocin or serine hydroxamate (SHX). Both chemicals are known to induce (p)ppGpp production by inhibiting the isoleucyl-tRNA synthetase (in *E*. *coli*^49^ and in various *Streptococcus*^27,50,51^ and *Staphylococcus*^52^ species) or the seryl-tRNA synthetase (i.a. in *T*. *thermophilus*, *B*. *subtilis*, *E*. *coli*^53,54^), respectively, and thus by “simulating” starvation for the respective amino acid^55^. Full medium (LB) was used as a control to compare the magnitude of the induced change in promoter activity. Nile red at a concentration of 20 µg/ml was utilized to monitor growth for normalization of the signal and to account for variations in the growth rate. Interestingly, adding 1 µg/ml of both chemicals seemed to have a very weak positive effect on the activity of the *comEA/EC* promoter. However, the addition of both mupirocin and SHX at a concentration of 100 µg/ml and 10 µg/ml, respectively, caused a significant repressing effect on the promoter in comparison to the promoter activity of cells incubated in pure MM (Fig. 7). In both cases, the relative promoter activity was decreased by approximately 75%. As expected, the normalized signal for cells grown in full medium remained low which correlates well with the decrease in transformability of the bacteria in LB. This data supports a hypothetical model where a high increase in intracellular (p)ppGpp production would cause a strong downregulation of numerous genes as is typical for the stringent response, including the competence-related genes as well, and would shift the cellular resources towards stress survival. This hypothetical model could also explain why auxotrophy - that comes about with a vast accumulation of (p)ppGpp - inhibits competence development and how amino acids supplemented to the growth medium could exert their effect on natural transformation in *M*. *luteus*.

## Discussion

This study represents the first detailed report about the regulation of development of competence for natural transformation in a member of the Gram-positive phylum *Actinobacteria*. Competence is only seldom a constitutive trait but rather in most cases underlies complex regulation, being influenced by different environmental cues^12^. The environmental triggers that induce the competent state and the influencing variables that contribute to the fine-tuning of the transformation apparatus vary among different bacterial species. As an example, a recent study by Moreno-Gámez *et al*.^10^ demonstrated that natural transformation in *S*. *pneumoniae* is dependent on cell density, external pH, antibiotic-induced stress and cell history and that these cues are integrated via a quorum sensing mechanism. Growth phase, nutritional state, availability of particular carbon sources, DNA damage and mutational burden have also been implicated in the induction of competence^1,56^. In our previous work, we provided evidence that the transformation machinery in *M*. *luteus* is a target of a complex regulation that is affected by the growth phase of the bacteria and the nutrient availability in the growth medium^38^. In this report, we were able to narrow down the search for inducing cues and to identify amino acid starvation as an inducer of competence. On the transcriptional level, it appeared that the expression of the late competence-related gene *comEA/EC* was inversely correlated to the concentration of amino acids in the growth medium with the highest *comEA/EC* expression achieved at the lowest concentration of amino acids. In terms of transformability, a similar connection was observed. However, experiments with different amounts of peptone or amino acids mixtures revealed that the transformation frequency did not change gradually with an increasing concentration of amino acids as seemed to be the case on the transcriptional level. In fact, the transformation frequency decreased abruptly above a certain threshold concentration of amino acids in the growth medium (Fig. 1). Low amino acid concentrations may even exert a weak positive effect on transformability. However, the latter effect could also have its reason in the protocol that was used for the execution of the transformation frequency assays. Since the transformability was assessed after 20 hours of incubation in the respective media, it seems plausible that low amounts of amino acids get used up in this period, leading to an amino acid-exhausted medium so that cells transition into a “hunger state” at the time of performance of the assay. This would be similar to a transition from full medium to MM which is known to trigger competence development in *M*. *luteus*^38^. However, it is important to mention that the performed transformation frequency assays are not able to account for all the factors that might affect the transformation frequency of the cell population. Different supplementations can exert different effects on the growth rate of the bacteria leading to variations in the growth phase and the cell density of the tested cultures. Trying to keep the growth phase constant, however, would also affect the other parameters such as incubation time and optical density. This makes it extremely difficult to follow the effect of one single parameter. A faster, more robust and more sensitive method for measuring transformability would greatly enhance the investigation into these questions. Still, by performing many different assays at various time points and growth stages and under differing conditions, we were able to observe a similar trend each time and to pinpoint amino acid starvation as a trigger of competence.

In addition, in this report, we also demonstrated that auxotrophic conditions completely inhibit natural transformation in *M*. *luteus* (Fig. 2). It seems logical to assume that starving cells would turn down their overall protein expression due to an inability to translate transcripts when a certain amino acid is missing. However, this is not a response affecting all genes. In order for the bacteria to survive stress conditions, they are dependent on many functions connected to amino acid and carbohydrate transport and metabolism, coenzyme production, defense mechanisms, and energy conservation. Indeed, transcriptome sequencing of the leucine auxotrophic mutant (strain ∆08730:k) indicated that during growth under auxotrophic conditions a large number of genes is highly upregulated (Supplementary Fig. S4). In this regard, our data suggest that natural transformation is not among the strategies that the bacteria use to try and overcome metabolic stress of this kind. This is an important finding because the evolutionary function of natural transformation is still actively debated in the scientific literature with two of the main hypotheses proposing a nutritional role or a role as a DNA repair template for the internalized DNA^11^.

The investigation into the modulatory function of each individual amino acid also revealed an interesting pattern. Supplementation with several of the amino acids, including histidine, serine, valine, isoleucine, methionine, cysteine, and threonine, caused strong repression of the promoter of *comEA/EC* (Fig. 4). Some of these amino acids also impaired the growth of the bacteria when added to our defined MM. Other studies have also observed a similar effect of supplemented amino acids on growth. Mostly, such observations are explained with feedback inhibition of metabolic pathways that are shared with the biosynthesis pathways of other amino acids^45,57^. This way, supplementation with a single amino acid in excess could, in theory, cause a temporary auxotrophy for another amino acid, which would result in a reduced growth rate and also in a reduced transformability. As an example, in *M*. *luteus*, the pathway for valine biosynthesis shares several common reactions with the pathway for the production of leucine. Thus, supplemented valine might inhibit the production of leucine and might cause starvation for this amino acid which in turn may explain the decrease in the transcription of the *comEA/EC* gene. Furthermore, this hypothesis would also provide an explanation for the fact that both valine and isoleucine reduce transformability, whereas addition of all three BCAAs to the growth medium circumvents this repression (Fig. 5A,B).

In order for the bacteria to be able to respond to environmental stimuli, they have evolved many different systems to perceive changes in their surroundings. In the context of natural transformation, the most common mechanism is quorum sensing. In many competent microorganisms, quorum sensing is responsible for triggering competence after the onset of the appropriate conditions. However, since previous preliminary work in our group (unpublished) had not revealed competence inducing peptides or other components of a quorum sensing system in *M*. *luteus*, we decided to look for an alternative response that may translate the “hunger” for amino acids into a state of competence. Typically, the stringent response is the most common and widely distributed response of bacteria when they face nutrient limitation or other stress factors. It is accompanied by a massive change in the transcription profile of the cells and it interferes with a vast majority of metabolic processes. Our study for the first time has targeted genetic determinants of the putative stringent response system of *M*. *luteus* (Fig. 6). It represents one of the very few studies that demonstrate a connection between the stringent response and natural transformation. Furthermore, to the best of our knowledge, this is the first work that provides evidence for such a link in an organism that does not possess a homolog of the GTP-sensing repressor CodY.

In accordance with our data, we propose a regulation model where the intracellular (p)ppGpp would regulate the transformability of the cells in such a manner that the concentration-response curve would have a bell-shaped form. More precisely, during transition from full medium to minimal medium, the alarmone would accumulate in the cells due to the lack of amino acids in MM. Under these conditions, the bacteria would increase their own amino acid biosynthesis which in turn would cause oscillations in the concentration of (p)ppGpp. When equilibrium sets in, the cells would reach their maximum transformability. Lower amounts of the alarmone, caused for example by supplementation with external amino acids, would decrease the transformation frequency. On the other hand, higher amounts due to auxotrophy would have a similar effect. This model may also provide an explanation for the apparent bidirectional effect of added mupirocin or SHX to the growth medium on the relative promoter activity of *comEA/EC* (Fig. 7A). The results from these experiments indicated that the transcription of the competence-related gene is specifically regulated by the concentration of the alarmone. Future measurements of the intracellular (p)ppGpp concentration in the different mutant strains would shed more light on this topic. In addition, more research is still needed to address certain issues associated with such a hypothesis. For example, while the RelA-SpoT homologue (RSH) family of bifunctional proteins is well studied, the exact type of nutrient limitation that activates enzymes of the SAS family remains unclear. This makes it difficult to draw conclusions about all of the nutritional signals that contribute to the production of (p)ppGpp. Furthermore, a recent discovery of a possible direct inhibiting interaction between RelA and the late competence ATPase ComGA in *B*. *subtilis* on a protein level^58^ demonstrates once again the convoluted control mechanisms governing the stringent response. Therefore, it is important to mention that our hypothetical model represents only the first step in unravelling the regulation of competence development in *M*. *luteus*, which could serve as a model also for other members of the *Actinobacteria*.

## Methods

### Bacterial strains and growth conditions

All strains used in this study are listed in Table 2. All mutant strains are derivatives of *Micrococcus luteus* trpE16, which is a tryptophan auxotroph of the strain “*Micrococcus lysodeikticus*” ISU^59^. Liquid cultures were incubated in baffled flasks with either lysogeny broth medium (LB) or glutamate minimal medium (MM) at 30 °C in a shaking incubator. LB was prepared according to the Lennox formulation^60^ and contained 10 g/L peptone, 5 g/L yeast extract, and 5 g/L NaCl. The pH was adjusted to 7.2 with HCl. MM was prepared according to Wolin and Naylor^40^ and consisted of 2 g/l K_2_HPO_4_, 1 g/l NH_4_Cl, 10 g/l sodium glutamate, 7 g/l glucose, 0.1 g/l MgSO_4_, 0.004 g/l FeSO_4_ and 0.002 g/l MnCl_2_. Unless otherwise specified, 0.1 mg/ml tryptophan was included to allow growth of the tryptophan auxotrophs. The pH of the MM was adjusted to 7.2 with HCl. For cultivation on solid plates, the liquid media were supplemented with 13 g/L agar. Strains carrying a kanamycin or a hygromycin resistance cassette were cultured in media supplemented with 60 µg/ml kanamycin sulfate or 100 µg/ml hygromycin B, respectively. For the transformation frequency assays, casein hydrolysate plates (CAH) containing 1% (w/v) sodium glutamate, 0.2% (w/v) K_2_HPO_4_, 0.1% (w/v) NH_4_Cl, 0.01% (w/v) MgSO_4_, 0.0004% (w/v) FeSO_4_, 0.0002% (w/v) MnCl_2_, 0.5% (w/v) tryptophan-free acid hydrolysed casein (EMD Millipore), 0.7% (w/v) glucose, and 1.3% (w/v) agar were used. For the amino acid supplementation experiments, we used the concentrations listed in Table 1 in accordance with the protocol used by Wolin and Naylor^40^. All amino acids were purchased from Formedium (Norfolk, UK) and were with ≥99% purity.

**Table 2:** List of all strains of *M*. *luteus* used in this study.

Strain	Genotype and relevant phenotype	Source
ATCC 27141	“*Micrococcus lysodeikticus*” ISU, Trp^+^	(Kloos, 1969)^4^
trpE16	*trpE16*, mutagenesis derivative of ATCC 27141, Trp^−^	(Kloos and Rose, 1970)^59^
Δ08730:k	trpE16 Δ08730::*kan*; Leu^−^; Kan^R^	This study
Δ03350:k	trpE16 Δ03350::*kan*; Kan^R^	This study
Δ*sas*:k	trpE16 Δ22200::*kan*; Kan^R^	This study
Δ*rel*:k	trpE16 Δ12840::*kan*; Kan^R^	This study
Δ*rel*:h	trpE16 Δ12840::*hyg*; Hyg^R^	This study
Δ*sas*:k Δ*rel*:h	trpE16 Δ22200::*kan* Δ12840::*hyg*; Hyg^R^; Kan^R^	This study
Δ*comEA*/*EC*:*lacZ*	trpE16 Δ*comEA/EC*::*lacZ-kan*; Kan^R^, LacZ expression	This study
Δ01920:tdT	trpE16 Δ01920::*tdT*-*kan*; Kan^R^, tdTomato expression	This study

### Construction of gene deletion mutants and reporter strains

All manipulations of the genome of *M*. *luteus* trpE16 were accomplished by using natural transformation and homologous recombination. For generating gene deletions, most of the coding sequence of the target gene was replaced by an antibiotic resistance cassette. Briefly, a linear DNA fragment consisting of approx. 1 kbp homology arms (chromosomal DNA regions upstream and downstream of the target gene) flanking either the Tn5 kanamycin resistance gene^61^ or the hygromycin resistance gene of the plasmid pSMT3-M^62^ (Addgene plasmid #26589) was first constructed. The assembly was carried out *in vitro* by mixing equimolar amounts of the three PCR-amplified fragments in a 20 µl Gibson Assembly reaction^63^. Competent cells of *M*. *luteus* trpE16 were directly transformed with the Gibson Assembly reaction and transformants were selected for on LB plates containing the appropriate antibiotics. For the construction of the reporter strain Δ*comEA*/*EC*:*lacZ*, a similar approach was used. However, an additional fourth PCR-amplified fragment was added to the assembly mixture to insert the reporter protein between the upstream homology arm and the antibiotic resistance cassette. The upstream homology arm of the exchange allele ended 1 bp upstream of the start codon of the target gene so that the expression of the reporter protein remained under the control of the native *comEA/EC* promoter. The coding sequence of *lacZ* from *E*. *coli* was amplified from the previously constructed vector pMKO^64^.

### Transformation frequency assay

For transformation frequency assays, cells from an overnight culture grown in complete medium were transferred to 20 ml MM (with the respective supplementation if needed) to give an initial optical density of 0.2 measured by absorbance at 600 nm. After 20 hours of incubation at 30 °C in a shaking incubator (180 rpm), the OD_600_ was determined to serve as a marker for the total cell count and 2 ml of the MM cultures were pelleted by centrifugation at 11000 × *g* for 8 min at 4 °C. The supernatant was discarded and the cells were resuspended in 1 ml transformation buffer (100 mM CaCl_2_, 50 mM Tris-HCl, pH 7.0). Next, 300 ng of a plasmid carrying the wild-type *trpE* allele from wild-type *M*. *luteus* ATCC 27141 (pJET-trpE, constructed by cloning of the wild-type *trpE* gene in pJET in *E*. *coli*) was added to the cell suspension. If internalized by natural transformation, this DNA is suited to allow conversion of trpE16 tryptophan auxotrophic cells back to prototrophy. Following incubation for 30 min at 30 °C in a shaking incubator (180 rpm), the transformation reaction was stopped by placing the cells on ice. The cell suspensions were sonicated for 1 min with 30% amplitude and 0.25 duty cycle to gently disrupt cell aggregates before plating on CAH agar plates to score transformants and on LB plates to determine the total viable cell counts. Control reactions performed without the addition of DNA delivered the rate of spontaneous reversion to prototrophy which determined the detection limit of the assay.

### Promoter activity assay

Precultures of the transcriptional reporter strain Δ*comEA*/*EC*:*lacZ* were grown overnight in LB. The following day, aliquots of the cells were harvested and washed with Tris-HCl (pH 7.2) and 20 µl aliquots were transferred to a 96-well microtiter plate containing 180 µl of medium per well (with respective supplementation if needed) to give an initial optical density of 0.05 measured by absorbance at 600 nm. For the measurement of LacZ activity, β-galactosidase substrate 4-methylumbelliferyl β-D-galactopyranoside (Sigma-Aldrich, USA) was added to each well at a final concentration of 100 µg/ml. Kinetic measurements were performed by incubating the cells in a FLUOstar Omega (BMG LABTECH, Germany) microplate reader at 30 °C for 96 hours and measuring MUG fluorescence every 10 minutes at 355/460 nm. Nile red was also used at a concentration of 20 µg/ml as a proxy for bacterial growth during incubation. Its fluorescence at 544/620 nm was used for normalization of the MUG signal (by dividing the MUG fluorescence values by the NR fluorescence values at each time point). The wild-type strain of *M*. *luteus* was incubated with and without a substrate to establish the background fluorescence. To determine the relative activity of the promoter of *comEA/EC*, a four parameter logistic function was fitted to the measured kinetic curves. The slope of the curve was calculated and was used for comparison. A detailed method validation is described in Supplementary Information.

### Quantitative real-time polymerase chain reaction

In this study, quantitative real-time PCR (qRT-PCR) was performed to measure the relative expression of three competence-related genes (*tadA1*, *tadA2*, *comEA*) in different strains and under different growth conditions. For this purpose, RNA was first isolated from the tested cells using the ZR Fungal/Bacterial RNA MiniPrep kit (Zymo Research, USA). An additional DNase treatment was performed with the TURBO DNA-free Kit (Thermo Fisher Scientific, USA) to minimize the amount of genomic DNA left in the samples. Next, RNA was reverse transcribed into cDNA by using the iScript cDNA synthesis kit (Bio-Rad Laboratories, USA) in a 20 µl reaction containing 1 µg RNA. Following a 1:5 dilution of the cDNA in DNase-free water, the qRT-PCR was performed with the SsoAdvanced Universal SYBR Green Supermix (Bio-Rad Laboratories, USA) on a CFX96 Touch Real-Time PCR Detection System (Bio-Rad Laboratories, USA). Non-template controls and “no reverse transcriptase” reactions were included to validate the results. The cycling parameters were 30 sec at 98 °C followed by 40 cycles of 15 sec at 95 °C and 20 sec at 60 °C, with a final extension step of 1 min at 55 °C. The _∆∆_Ct method was used for evaluation of the data^65^. Mlut_08120 which encodes the “A” region of the F_o_ subunit of a conserved ATP synthase was used as an internal reference gene and the wild-type strain grown in MM for 20 hours was used as a reference treatment. Two biological replicates were performed for each tested condition. Three technical replicates were performed for each investigated gene. The sequences of the used primers are provided in Supplementary Table S2 (Supplementary Information).

## Supplementary information

Supplementary information

## Data Availability

The datasets generated during and/or analysed during the current study are available from the corresponding author on reasonable request.

## References

[1] Johnston C, Martin B, Fichant G, Polard P, Claverys JP (2014). Bacterial transformation: distribution, shared mechanisms and divergent control. Nat. Rev. Microbiol..

[2] Ochman H, Lawrence JG, Groisman EA (2000). Lateral gene transfer and the nature of bacterial innovation. Nature.

[3] Croucher NJ (2011). Rapid pneumococcal evolution in response to clinical interventions. Science.

[4] Kloos WE (1969). Factors affecting transformation of *Micrococcus lysodeikticus*. J. Bacteriol..

[5] Kloos WE (1969). Transformation of *Micrococcus lysodeikticus* by various members of the family *Micrococcaceae*. J. Gen. Microbiol..

[6] Kloos WE, Schultes LM (1969). Transformation in *Micrococcus lysodeikticus*. J. Gen. Microbiol..

[7] Dubnau D (1999). DNA uptake in bacteria. Annu. Rev. Microbiol..

[8] Chen I, Dubnau D (2003). DNA transport during transformation. Front. Biosci..

[9] Chen I, Dubnau D (2004). DNA uptake during bacterial transformation. Nat. Rev. Microbiol..

[10] Moreno-Gámez S (2017). Quorum sensing integrates environmental cues, cell density and cell history to control bacterial competence. Nat. Commun..

[11] Claverys JP, Prudhomme M, Martin B (2006). Induction of competence regulons as a general response to stress in Gram-positive bacteria. Annu. Rev. Microbiol..

[12] Seitz P, Blokesch M (2013). Cues and regulatory pathways involved in natural competence and transformation in pathogenic and environmental Gram-negative bacteria. FEMS Microbiol. Rev..

[13] Yamamoto S (2011). Identification of a chitin-induced small RNA that regulates translation of the *tfoX* gene, encoding a positive regulator of natural competence in *Vibrio cholerae*. J. Bacteriol..

[14] Biørnstad TJ, Håvarstein LS (2011). ClpC acts as a negative regulator of competence in *Streptococcus thermophilus*. Microbiology (Reading, Engl.).

[15] Sung CK, Morrison DA (2005). Two distinct functions of ComW in stabilization and activation of the alternative sigma factor ComX in *Streptococcus pneumoniae*. J. Bacteriol..

[16] Redfield RJ (1991). *sxy-1*, a *Haemophilus influenzae* mutation causing greatly enhanced spontaneous competence. J. Bacteriol..

[17] Prudhomme M, Attaiech L, Sanchez G, Martin B, Claverys JP (2006). Antibiotic stress induces genetic transformability in the human pathogen *Streptococcus pneumoniae*. Science.

[18] Charpentier X, Kay E, Schneider D, Shuman HA (2011). Antibiotics and UV radiation induce competence for natural transformation in *Legionella pneumophila*. J. Bacteriol..

[19] Wilson GA, Bott KF (1968). Nutritional factors influencing the development of competence in the *Bacillus subtilis* transformation system. J. Bacteriol..

[20] Hauryliuk V, Atkinson GC, Murakami KS, Tenson T, Gerdes K (2015). Recent functional insights into the role of (p)ppGpp in bacterial physiology. Nat. Rev. Microbiol..

[21] Dalebroux ZD, Swanson MS (2012). ppGpp: magic beyond RNA polymerase. Nat. Rev. Microbiol..

[22] Potrykus K, Cashel M (2008). (p)ppGpp: still magical?. Annu. Rev. Microbiol..

[23] Atkinson GC, Tenson T, Hauryliuk V (2011). The RelA/SpoT homolog (RSH) superfamily: distribution and functional evolution of ppGpp synthetases and hydrolases across the tree of life. PLoS ONE.

[24] Ito D (2012). Enzymatic and molecular characterization of Arabidopsis ppGpp pyrophosphohydrolase, AtNUDX26. Biosci. Biotechnol. Biochem..

[25] Ooga T (2009). Degradation of ppGpp by nudix pyrophosphatase modulates the transition of growth phase in the bacterium *Thermus thermophilus*. J. Biol. Chem..

[26] Hamel E, Cashel M (1973). Role of guanine nucleotides in protein synthesis. Elongation factor G and guanosine 5′-triphosphate,3′-diphosphate. Proc. Natl. Acad. Sci. USA.

[27] Lemos JA, Lin VK, Nascimento MM, Abranches J, Burne RA (2007). Three gene products govern (p)ppGpp production by *Streptococcus mutans*. Mol. Microbiol..

[28] Cao M (2002). Defining the *Bacillus subtilis* sigma(W) regulon: a comparative analysis of promoter consensus search, run-off transcription/macroarray analysis (ROMA), and transcriptional profiling approaches. J. Mol. Biol..

[29] Sun D (2010). A metazoan ortholog of SpoT hydrolyzes ppGpp and functions in starvation responses. Nat. Struct. Mol. Biol..

[30] Dalebroux ZD, Svensson SL, Gaynor EC, Swanson MS (2010). ppGpp conjures bacterial virulence. Microbiol. Mol. Biol. Rev..

[31] Geiger T (2012). The stringent response of *Staphylococcus aureus* and its impact on survival after phagocytosis through the induction of intracellular PSMs expression. PLoS Pathog..

[32] Poole K (2012). Bacterial stress responses as determinants of antimicrobial resistance. J. Antimicrob. Chemother..

[33] Maisonneuve E, Gerdes K (2014). Molecular mechanisms underlying bacterial persisters. Cell.

[34] Seaton K, Ahn SJ, Sagstetter AM, Burne RA (2011). A transcriptional regulator and ABC transporters link stress tolerance, (p)ppGpp, and genetic competence in *Streptococcus mutans*. J. Bacteriol..

[35] Seaton K, Ahn SJ, Burne RA (2015). Regulation of competence and gene expression in *Streptococcus mutans* by the RcrR transcriptional regulator. Mol. Oral Microbiol..

[36] Kaspar J, Kim JN, Ahn SJ, Burne RA (2016). An essential role for (p)ppGpp in the integration of stress tolerance, peptide signaling, and competence development in *Streptococcus mutans*. Front. Microbiol..

[37] Inaoka T, Ochi K (2002). RelA protein is involved in induction of genetic competence in certain *Bacillus subtilis* strains by moderating the level of intracellular GTP. J. Bacteriol..

[38] Angelov A (2015). Novel Flp pilus biogenesis-dependent natural transformation. Front. Microbiol..

[39] Sezonov G, Joseleau-Petit D, D’Ari R (2007). *Escherichia coli* physiology in Luria-Bertani broth. J. Bacteriol..

[40] Wolin HL, Naylor HB (1957). Basic nutritional requirements of *Micrococcus lysodeikticus*. J. Bacteriol..

[41] Surger MJ, Angelov A, Stier P, Übelacker M, Liebl W (2018). Impact of branched-chain amino acid catabolism on fatty acid and alkene biosynthesis in *Micrococcus luteus*. Front. Microbiol..

[42] Zhou Y, Huang H, Zhou P, Xie J (2012). Molecular mechanisms underlying the function diversity of transcriptional factor IclR family. Cell Signal..

[43] Brune I (2007). The IclR-type transcriptional repressor LtbR regulates the expression of leucine and tryptophan biosynthesis genes in the amino acid producer *Corynebacterium glutamicum*. J. Bacteriol..

[44] Liu M, Prakash C, Nauta A, Siezen RJ, Francke C (2012). Computational analysis of cysteine and methionine metabolism and its regulation in dairy starter and related bacteria. J. Bacteriol..

[45] Massey LK, Sokatch JR, Conrad RS (1976). Branched-chain amino acid catabolism in bacteria. Bacteriol. Rev..

[46] Kaiser JC, Omer S, Sheldon JR, Welch I, Heinrichs DE (2015). Role of BrnQ1 and BrnQ2 in branched-chain amino acid transport and virulence in *Staphylococcus aureus*. Infect. Immun..

[47] Stenz L (2011). The CodY pleiotropic repressor controls virulence in Gram-positive pathogens. FEMS Immunol. Med. Microbiol..

[48] Shivers RP, Sonenshein AL (2004). Activation of the *Bacillus subtilis* global regulator CodY by direct interaction with branched-chain amino acids. Mol. Microbiol..

[49] Hughes J, Mellows G (1978). Inhibition of isoleucyl-transfer ribonucleic acid synthetase in *Escherichia coli* by pseudomonic acid. Biochem. J..

[50] Mechold U, Malke H (1997). Characterization of the stringent and relaxed responses of *Streptococcus equisimilis*. J. Bacteriol..

[51] Whitehead KE, Webber GM, England RR (1998). Accumulation of ppGpp in *Streptococcus pyogenes* and *Streptococcus rattus* following amino acid starvation. FEMS Microbiol. Lett..

[52] Cassels R, Oliva B, Knowles D (1995). Occurrence of the regulatory nucleotides ppGpp and pppGpp following induction of the stringent response in staphylococci. J. Bacteriol..

[53] Kasai K (2006). Physiological analysis of the stringent response elicited in an extreme thermophilic bacterium, *Thermus thermophilus*. J. Bacteriol..

[54] Pizer LI, Merlie JP (1973). Effect of serine hydroxamate on phospholipid synthesis in. Escherichia coli. J. Bacteriol..

[55] Tosa T, Pizer LI (1971). Biochemical bases for the antimetabolite action of L-serine hydroxamate. J. Bacteriol..

[56] Gagne AL (2013). Competence in *Streptococcus pneumoniae* is a response to an increasing mutational burden. PLoS ONE.

[57] Traxler MF (2008). The global, ppGpp-mediated stringent response to amino acid starvation in *Escherichia coli*. Mol. Microbiol..

[58] Hahn J, Tanner AW, Carabetta VJ, Cristea IM, Dubnau D (2015). ComGA-RelA interaction and persistence in the *Bacillus subtilis* K-state. Mol. Microbiol..

[59] Kloos WE, Rose NE (1970). Transformation mapping of tryptophan loci in *Micrococcus luteus*. Genetics.

[60] Lennox ES (1955). Transduction of linked genetic characters of the host by bacteriophage P1. Virology.

[61] Beck E, Ludwig G, Auerswald EA, Reiss B, Schaller H (1982). Nucleotide sequence and exact localization of the neomycin phosphotransferase gene from transposon Tn5. Gene.

[62] Carroll P (2010). Sensitive detection of gene expression in mycobacteria under replicating and non-replicating conditions using optimized far-red reporters. PLoS ONE.

[63] Gibson DG (2009). Enzymatic assembly of DNA molecules up to several hundred kilobases. Nat. Methods.

[64] Angelov A, Li H, Geissler A, Leis B, Liebl W (2013). Toxicity of indoxyl derivative accumulation in bacteria and its use as a new counterselection principle. Syst. Appl. Microbiol..

[65] Livak KJ, Schmittgen TD (2001). Analysis of relative gene expression data using real-time quantitative PCR and the 2(-Delta Delta C(T)) method. Methods.

