# Supplementary material for: Amino acids as nutritional factors and (p)ppGpp as an alarmone of the stringent response regulate natural transformation in Micrococcus luteus
Source: Sci Rep. 2019 Jul 30;9:11030. doi: 10.1038/s41598-019-47423-x (PMC6667448; doi:10.1038/s41598-019-47423-x)
Supplement: Supplementary file 1 — Supplementary information [file 41598_2019_47423_MOESM1_ESM.pdf]

Supplementary information

Lichev, A., Angelov, A., Cucurull, I., Liebl, W.

"Amino acids as nutritional factors and (p)ppGpp as an alarmone of the stringent response regulate natural transformation in *Micrococcus luteus*"

Supplementary methods

Validation of the promoter activity assay

For the investigation of the relative activity of the promoter of *comEA/EC*, we used the transcriptional reporter strain Δ*comEA*/*EC*:*lacZ* that expresses the full-length *E. coli lacZ* gene from the native *comEA/EC* promoter. Kinetic measurements were performed by utilizing the fluorogenic LacZ substrate 4-methylumbelliferyl-β-D-galactopyranoside (MUG) that has been reportedly used in similar experiments in Gram-positive[66,67] and Gram-negative bacteria, yeast and mammalian cells[68]. To validate the applicability of this approach for *M. luteus*, we first investigated the correlation between the measured MUG fluorescence signal resulting from incubation of the substrate with whole cells and the signal obtained from crude extracts. This served to verify that the kinetic measurements of the MUG fluorescence during bacterial growth accurately reflected the actual enzymatic LacZ activity inside of the cells. For this, the reporter strain Δ*comEA*/*EC*:*lacZ* was first inoculated in 200 µl of both LB and MM at different initial optical densities in a 96-well microtiter plate. For half of the replicates (n = 4), MUG was added at a concentration of 100 µg/ml. After incubating the cells for 22 hours at 30 °C and measuring MUG fluorescence every 10 min, the cells in the second half of the replicates (n = 4) that did not contain any substrate were rapidly lysed by the addition of 100 µg lysozyme. MUG was then added to the lysed cells at a concentration of 100 µg/ml and the fluorescence signal was measured every 1 min at 355/460 nm for 60 min. The kinetic curves resulting from the measured fluorescence signal during incubation of MUG with whole cells were evaluated by calculating the slope of the curves generated in the last 90 min of the measurements. For the cells demonstrating a very high LacZ activity, the fluorescing substrate was depleted within the 22 hours

resulting in a non linear kinetic curve during the last 90 min of incubation. Here, the linear range of the kinetic curves prior to substrate depletion was used for evaluation. The kinetic curves resulting from the measured fluorescence signal during incubation of MUG with lysed cells were evaluated by calculating the steepest slope within the linear range of the curves which corresponded to the maximal velocity (V_{max}) of the enzymatic reaction and was directly proportional to the enzyme concentration. The correlation between the signals arising from whole cells and crude extracts is depicted in Supplementary Figure S1. The wild-type strain of *M. luteus* was used as a control to monitor the background MUG fluorescence for the experimental setups with both whole and lysed cells.

Next, in order to be able to measure bacterial growth during longer incubations and to account for differences in cell counts during fluorescence measurements, we utilized the lipophilic stain Nile red at a concentration of 20 µg/ml that fluoresces upon binding to lipophilic substances. Due to strong aggregation of the bacterial cells during growth in a microtiter plate, measurements of the optical density were not suitable for this task. However, previous preliminary work in our group (unpublished) had revealed that incubation of bacterial cells in a microtiter plate with added Nile red resulted in a fluorescence signal resembling a typical growth curve. To validate the correlation between the Nile red fluorescence signal and the cell count, we also created a dilution series of a bacterial suspension with an initial OD_{600} of 10 (measured after sonication for mild aggregate disruption). Next, we added Nile red to each dilution at a concentration of 20 µg/ml. After incubating the cells with the dye for 5 min at 30 °C, the Nile red fluorescence was measured at 544/620 nm and was plotted against the calculated OD_{600} of each dilution (Supplementary Figure S1).

Fitting of a logistic model to the measured kinetic data for the *comEA/EC* promoter

To determine the relative activity of the promoter of *comEA/EC* under supplementation with different amino acids (Fig. 4), a four parameter logistic function was fitted to the measured kinetic curves after normalization by the fluorescence signal of Nile red. The slope of the fitted curve was calculated and was used for comparison. The following equation was used for the fitting of the logistic function:

Y=Bottom + (Top-Bottom)/(1+10^((LogIC50-X)*Slope)).

The calculated best-fit parameters, the standard error for the calculated parameters and the goodness of the fit are listed in Supplementary Table S1.

Supplementary references

66. Watson, A.L., Chiu, N.H.L. Fluorometric cell-based assay for β-galactosidase activity in probiotic Gram-positive bacterial cells - *Lactobacillus helveticus*. *J. Microbiol. Methods* **128,** 58-60 (2016).

67. Chiu, N.H.L., Watson, A.L. Measuring β-galactosidase activity in Gram-positive bacteria using a whole-cell assay with MUG as a fluorescent reporter. *Curr. Protoc. Toxicol.* **74,** 4.44.1-4.44.8 (2017).

68. Vidal-Aroca, F. *et al*. One-step high-throughput assay for quantitative detection of beta-galactosidase activity in intact gram-negative bacteria, yeast, and mammalian cells. *BioTechniques* **40**(4)**,** 433-4, 436, 438 passim (2006).

Supplementary figures

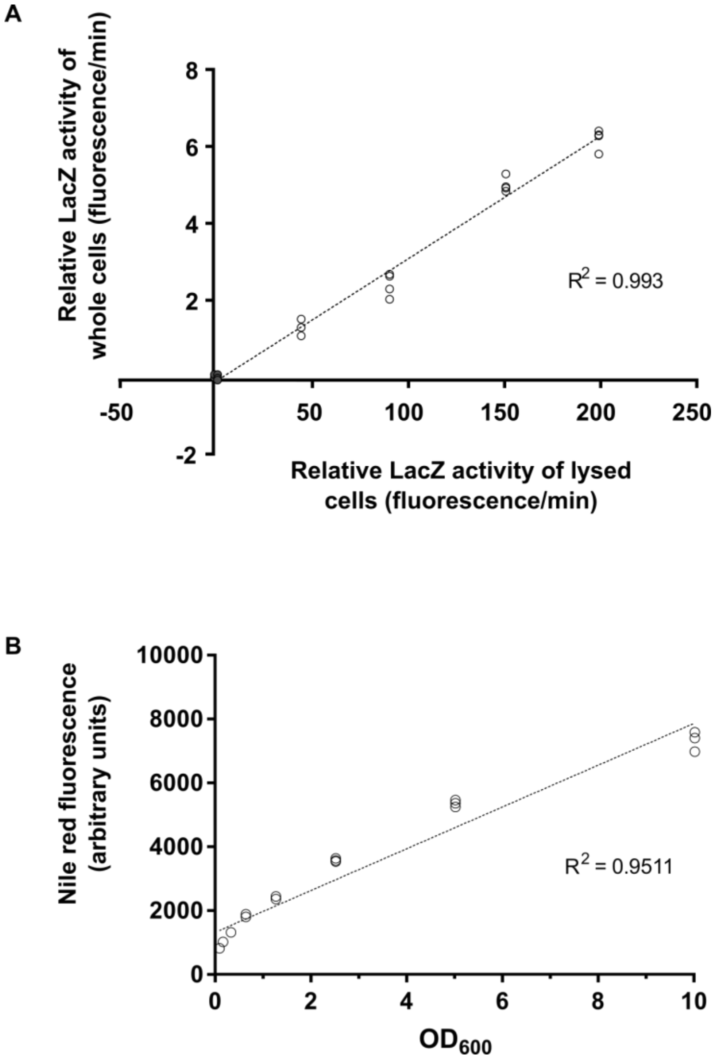

Supplementary Figure S1. Validation of the promoter activity assay.

(A) Correlation between the MUG fluorescence signal obtained during incubation of whole bacterial cells with MUG and the LacZ activity of the cells. Plotted on the x-axis are the mean slopes (n = 4) of the linear range of the kinetic curves resulting from incubation of lysed bacterial cells with MUG. Plotted on the y-axis are the slopes of the kinetic curves (n = 4) generated from incubation of whole cells with MUG (see Validation of the promoter activity assay, Supplementary methods, Additional file 1). The grey circles represent the background MUG fluorescence of whole and lysed cells of the reference strain *M. luteus* trpE16. (B) Correlation between Nile red fluorescence and optical density of a bacterial suspension. A dilution series of a bacterial suspension with OD_{600} ranging between 0.075 and 10 was incubated with 20 µg/ml Nile red for 5 min at 30 °C. The resulting fluorescence at 544/620 nm was plotted against the calculated optical density of each dilution (n = 3).

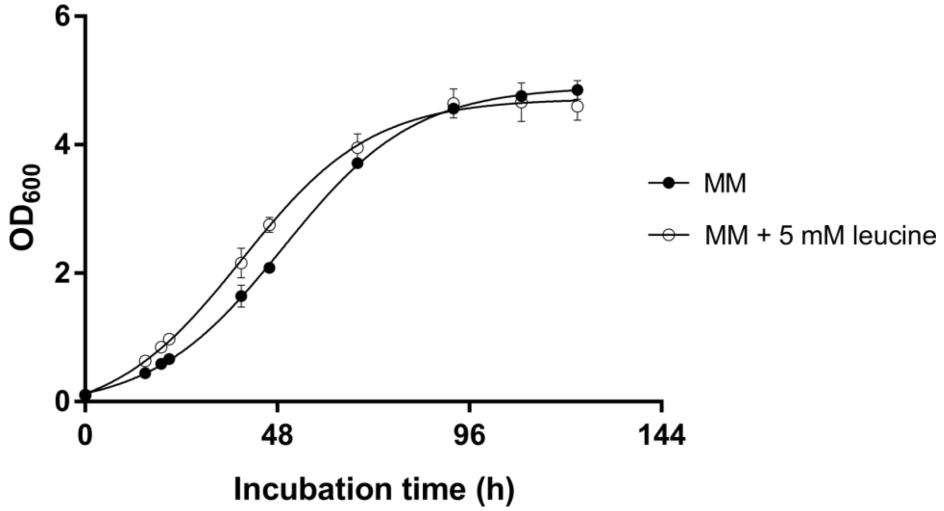

Supplementary Figure S2. Growth curve of the wild-type strain (trpE16) in MM supplemented with or without 5 mM leucine.

The wild-type strain of *M. luteus* (trpE16) was grown for 120 hours in MM (containing tryptophan) supplemented with (white dots) or without (black dots) 5 mM leucine. OD_{600} measurements were performed to assess the growth rate of the bacteria in the different cultures (n = 2). The dots represent the mean OD_{600} values. The error bars represent the SD of the replicates. The line represents a fitted logistic model to the measured data.

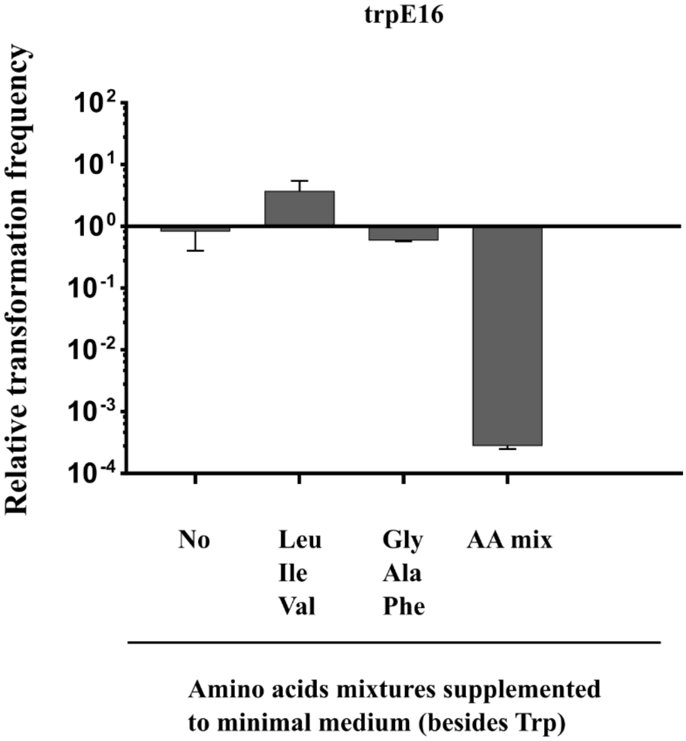

Supplementary Figure S3. Effect of different amino acids mixtures on the transformability of *M. luteus*. The wild-type strain of *M. luteus* (trpE16) was grown for 20 hours in MM (containing tryptophan) supplemented with BCAAs, a mixture of glycine, alanine and phenylalanine or a mixture of 15 amino acids (Table 1) at a total concentration of 15 mM. A transformation frequency assay revealed variations in the transformability of the differently supplemented cultures. Bars represent the mean transformation frequencies (n = 2) relative to the mean transformation frequency of the reference strain *M. luteus* trpE16 grown in MM for 20 hours, which was set to 1. The error bars indicate the SD of the biological replicates.

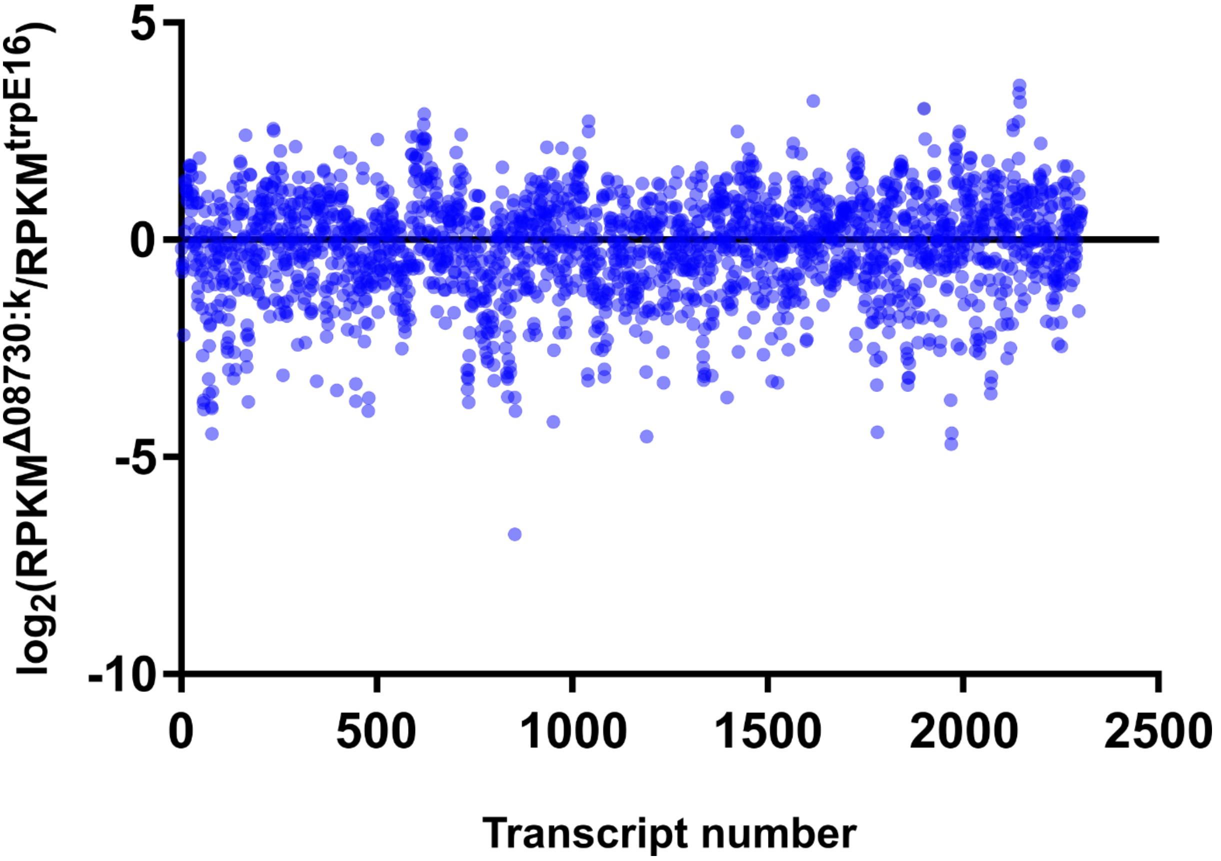

Supplementary Figure S4. Transcriptome sequencing of the leucine auxotrophic mutant Δ08730:k.

The leucine auxotrophic strain Δ08730:k was inoculated in MM (without leucine) at an initial OD_{600} of 0.2. Following an incubation for 20 hours, mRNA was isolated and sequenced. The wild-type trpE16 strain grown under the same conditions served as a reference strain. For each detected transcript, the log2 of the ratio of the RPKM values (reads per kilobase million) between the mutant strain and the wild-type strain was plotted (blue dots). Upregulated genes (in comparison to the reference strain) have values higher than 0. Downregulated genes have values lower than 0.

Supplementary tables

Supplementary Table S1. Best-fit parameters, standard error and goodness of the fit for the fitted logistic model to the measured kinetic data for the *comEA/EC* promoter under supplementation with different amino acids.

	Best-fit values						Std. Error					Goodness of Fit			
AA	Top	Bottom	LogIC50	**Slope**	IC50	Span	Top	Bottom	LogIC50	Slope	Span	Degrees of Freedom	R square	Absolute Sum of Squares	Sy.x
Pro	12.89	1.223	1.055	**4.856**	11.34	11.67	0.22	0.41	0.02	1.16	0.47	347.00	0.69	3754.00	3.29
MM	13.84	1.188	1.146	**3.661**	14.01	12.65	0.16	0.30	0.02	0.52	0.35	347.00	0.84	1942.00	2.37
Asn	16.02	1.205	1.161	**3.505**	14.5	14.81	0.19	0.34	0.02	0.48	0.40	347.00	0.84	2568.00	2.72
Leu	11.8	1.201	1.177	**3.462**	15.04	10.59	0.21	0.38	0.03	0.74	0.45	347.00	0.68	3232.00	3.05
Arg	10.57	1.148	1.226	**3.2**	16.84	9.424	0.06	0.11	0.01	0.22	0.13	347.00	0.95	272.90	0.89
Phe	12.19	1.11	1.016	**3.002**	10.38	11.08	0.14	0.31	0.02	0.40	0.35	347.00	0.82	1452.00	2.05
Gln	8.997	1.091	1.28	**2.831**	19.06	7.906	0.06	0.11	0.01	0.21	0.13	347.00	0.94	259.50	0.86
Ala	11.42	1.351	1.468	**2.627**	29.37	10.07	0.18	0.27	0.03	0.40	0.34	347.00	0.78	1897.00	2.34
Lys	9.975	1.212	1.367	**2.62**	23.27	8.763	0.09	0.15	0.02	0.25	0.19	347.00	0.90	546.80	1.26
Gly	13.86	1.286	1.396	**2.208**	24.88	12.58	0.17	0.28	0.03	0.24	0.35	347.00	0.86	1649.00	2.18
Trp	7.834	1	1.493	**2.201**	31.11	6.834	0.05	0.07	0.01	0.12	0.09	347.00	0.96	128.60	0.61
Glu	9.522	1.26	2.152	**2.047**	141.8	8.262	0.12	0.10	0.02	0.17	0.16	347.00	0.91	447.40	1.14
His	10.4	1.122	2.251	**1.296**	178.2	9.279	0.12	0.09	0.02	0.07	0.17	347.00	0.95	259.10	0.86
Ser	9.485	1.195	2.475	**0.9286**	298.6	8.29	0.15	0.08	0.03	0.05	0.19	347.00	0.96	122.80	0.60
Val	11.17	1.037	3.476	**0.8357**	2991	10.13	0.49	0.04	0.05	0.04	0.51	347.00	0.98	42.33	0.35
Ile	5.159	0.9916	3.088	**0.6946**	1224	4.167	0.11	0.02	0.03	0.02	0.12	347.00	0.99	4.99	0.12
Met	11.98	1.069	3.789	**0.6515**	6150	10.91	1.10	0.04	0.13	0.04	1.13	347.00	0.97	37.99	0.33
Cys	2.105	0.9761	2.858	**0.3717**	720.9	1.129	0.44	0.14	0.76	0.20	0.57	347.00	0.52	16.99	0.22
Thr	4.028	0.6941	3.438	**0.2858**	2738	3.334	0.78	0.15	0.60	0.07	0.92	347.00	0.93	6.81	0.14

Supplementary Table S2. Sequences of the oligonucleotides used for the performance of the qRT-PCR experiments described in this work.

**Primer**	**Sequence**	**Target gene**
q08120.F	TCGTGCTGCTGAACAACC	Mlut_08120
q08120.R	CAGATCGCGTAGACGATGAG	Mlut_08120
q12450.F	CCACGGCGGAGAAGATCAT	*comEA/EC*
q12450.R	GTTCACCACGAGACGAGGT	*comEA/EC*
q07500.F	GGCGGTTCCCTCACTCCGAC	*tadA1*
q07500.R	GGCCGAAGCCAGGCAGTTGAGC	*tadA1*
q18190.F	GGGCAAGACCACGCTGCTCTC	*tadA2*
q18190.R	ACCACGTGCGGGTGATCGG	*tadA2*
